# Sociodemographic and clinical determinants of in-facility case fatality rate for 938 adult Ebola patients treated at Sierra Leone Ebola treatment center

**DOI:** 10.1186/s12879-020-04994-9

**Published:** 2020-04-21

**Authors:** Jia Bainga Kangbai, Christian Heumann, Michael Hoelscher, Foday Sahr, Guenter Froeschl

**Affiliations:** 1 Center for International Health, University of Munich (LMU), Munich, Germany; 2 Department of Environmental Health Sciences, School of Community Health Sciences, Njala University, Bo, Sierra Leone; 3 Department of Statistics, University of Munich (LMU), Munich, Germany; 4 Division of Infectious Diseases and Tropical Medicine, University Hospital, LMU, Munich, Germany; 5 Department of Microbiology, College of Medicine and Allied Health Sciences, University of Sierra Leone, Freetown, Sierra Leone; 6 34 Military Hospital, Wilberforce, Sierra Leone

**Keywords:** Ebola, Ebola treatment center, Treatment outcome, Case fatality rate, Sierra Leone

## Abstract

**Background:**

The 2013–2016 West Africa Ebola Virus Disease (EVD) outbreak recorded the highest incidence and mortality since the discovery of the virus in Zaire in 1976; with more than 28,000 probable and confirmed EVD cases and 11,000 deaths. Studies relating to previous outbreaks usually involved small sample sizes. In this study we are set to identify those sociodemographic and clinical features that predict in-facility mortality among EVD patients using a large sample size.

**Methods:**

We analysed the anonymized medical records of 938 laboratory-confirmed EVD patients 15 years old and above who received treatment at The 34 Military Hospital and The Police Training School EVD Treatment Centers in Sierra Leone in the period June 2014 to April 2015. We used both univariable and multivariable logistic regression to determine the predictors for in-facility mortality of these patients based on their sociodemographic and clinical characteristics.

**Results:**

The median age of the EVD cases was 33 years (interquartile range = 25 to 40 years). The majority of the EVD cases were male (59.0%) and had secondary level education (79.3%). We reported a low overall in-facility case fatality rate of 26.4%. The associations between case fatality rates and EVD patients who reported fever, abdominal pain, cough, diarrhoea, vomiting, fatigue, haemorrhage, dysphagia, conjunctival injection, dyspnea, and skin rash at the time of admission were all statistically significant (*p* <  0.05). Our preferred model with the age group 65 years and above alongside the following clinical symptoms; diarrhoea, vomiting, fatigue, dysphagia, conjunctival injection, dyspnea and cough produced a receiver operating characteristic (ROC) curve with an AUC (area under the curve) value of 0.93.

**Conclusions:**

We constructed a simple model that can be optimally used alongside other rapid EVD diagnostic tools to identify EVD in-facility treatment mortality predictors based on the sociodemographic characteristics and clinical symptoms of adult EVD patients. We also reported low EVD cases among patients with secondary and tertiary education. These subpopulations of our patients who are generally informed about the signs and symptoms of EVD, alongside our treatment regimen may have been responsible for our comparatively lower case fatality rate.

## Background

More than 28,000 probable and confirmed Ebola Virus Disease (EVD) cases and 11,000 EVD-related deaths [1] were documented in the 2013–2016 West Africa outbreak; the highest prevalence and mortality since the discovery of the Ebola Virus Disease (EVD) in Zaire in 1976 [2]. The pathogen responsible for the West African EVD outbreak was different from those of previous outbreaks in the Democratic Republic of Congo and Gabon [3]. Sierra Leone recorded its first EVD case in May 2014 and had the highest burden of the disease (14,121 EVD cases, 3955 EVD-related deaths) during the outbreak [4]. The gender rather than the sex of a person plays an important role in the transmission and vulnerability to EVD infection. Sierra Leone’s first EVD case was a woman [5]. Several factors including the mode of acquisition of Ebola Virus determines the incubation period for EVD; direct Ebola Virus acquisition may lead to shorter incubation period [6, 7]. Several clinical symptoms of EVD have being identified; Barry et al. reported asthenia (80%), fever (72%), vomiting (60%), diarrhea (34%),myalgia (23%) as common clinical signs of EVD infection alongside headache, general body ache, rash and haemorrhagic diathesis [8]. Different types of Case Fatality Rates (CFR) for the 2013–2016 EVD outbreak have been reported. The CFR reported by Haaskjold Y et al. for a mixed cohort of EVD cases treated in Moyamba district in Sierra Leone during the 2013–2016 EVD outbreak was 40% [9]. The CFR of confirmed EVD cases with clinical outcomes for Sierra Leone, Guinea and Liberia were 68.9% (62.1–74.5%), 65.7% (61.4–69.5%), and 61.4% (55.9–67.3%) respectively [10]. The WHO Ebola Response Team reported similar CFR (70.8%) among EVD patients who reported specific haemorrhagic symptoms and “unexplained bleeding” [11] during the 2013–2016 EVD. Several organs and systems are generally affected during EVD infection. Schieffelin et al., discovered evidence of liver damage in both deceased and surviving EVD patients in Sierra Leone [12]. Another Sierra Leone study in 2014 recorded a low number of EVD patients with confusion and conjunctivitis [13]. Several factors have been associated for the different CFRs during EVD outbreak. Generally, predictors of higher CFR are age [12–14], diarrhoea, conjunctivitis [12–15] and high Ebola Virus viremia [12, 13, 16]. The CFR values were also varied according to the patient’s occupational status. Dallatomasina S, et al. recorded a higher (68%) CFR among health workers compared to other occupation (52%, *p* = 0.05) [17].

Previous studies relating to EVD infection were usually limited by their small sample sizes. Majority of the 56 laboratory-confirmed EVD cases in one Ugandan study had non-bloody diarrhoea (81%), severe headache (81%), and asthenia (77%) [18]. The CFR for 62 positive EVD patients treated at Moyamba Ebola Treatment Center in Sierra Leone during the 2013–2016 EVD outbreak was 68.9% [19].

In this investigation, we report on the factors associated with the treatment outcomes (CFR) of 938 laboratory - confirmed EVD cases that were treated by military personnel attached to The 34 Military Hospital and The Police Training School ETCs during the 2013–2016 outbreak in Sierra Leone. Our aim is to use our dataset to describe the clinical and sociodemographic determinants for EVD case treatment outcomes and to construct a model that can best predict EVD in-facility CFR using the clinical and sociodemographic characteristics of these patients.

## Methods

### Study design

We analysed the anonymized medical records of 938 laboratory-confirmed EVD patients who are 15 years of age and above who received treatment at The 34 Military Hospital and The Police Training School ETCs in Sierra Leone from June 2014 to April 2015. The period of June 2014 to April 2015 was the peak of the Ebola outbreak in Sierra Leone. A laboratory-confirmed EVD patient is defined as an ill person whose full blood, serum, or plasma specimen has been tested positive by quantitative reverse transcriptase polymerase chain reaction (RT-PCR) assay. All laboratory confirmatory tests on suspected EVD patients were done at the National Public Health Laboratory based at Lakkah in Freetown. The medical records of these EVD patients included their clinical symptoms and sociodemographic characteristics. Data clerks attached to The 34 Military Hospital and The Police Training School first collected these data on hard copies of Case Report Form (CRF) at the time of admission of these EVD patients and later converted them to digital form. We later used Microsoft Excel [20] for both descriptive and model-based data analysis.

### Study area

During the 2013–2016 Ebola outbreak, some government referral hospitals and district health centers, as well as private hospitals and clinics managed by foreign organisations inside Sierra Leone served as either an ETC or an Ebola Holding Centers (EHC). The 34 Military Hospital which provided data for this study, operated two geographically different ETCs three kilometers apart; The 34 Military Hospital and The Police Training School. The 34 Military Hospital ETC started EVD treatment in June 2014 but as the outbreak progresses and the facility became over burden with EVD cases, they extended their operation by opening The Police Training School center in August 2014; the two ETCs closed operation in April 2015. Both centers served the Western Area and Western Urban populations and were managed by medical personnel attached to the 34th Military Battalion of the Sierra Leone Armed Forces (SLAF). At the time of 2013–2016 EVD outbreak, The 34 Military Hospital was headed by a Brigadier Surgeon General and was assisted by medical doctors and paramedics. The 34 Military Hospital ETC accommodated 30 confirmed EVD cases and 20 suspected EVD cases awaiting laboratory test results while The Police Training School had 20 bed spaces to treat EVD cases; both ETCs were geographically separated by approximately 3 km.

### Ethics review

The Sierra Leone Ethics and Scientific Review Committee (Opinion Date March 29, 2017) and the Institutional Review Board at the Ludwig-Maximilians-Universität in Munich, Germany (Opinion No. LMU 17–582) approved this study.

### Data collection and processing

The datasets generated and analyzed during the current study are not publicly available due to patient confidentiality and the sensitive nature of this study. It is an aggregated datasets that is being protected by the Sierra Leone Ethics and Scientific Review Committee in order to protect the identity of the patients whose medical data were analyzed. EVD patients in this study had reported at The 34 Military Hospital and The Police Training School ETCs in Freetown either independently or through the National Ebola Response Center (NERC) surveillance system. Suspected EVD patients were first screened at the triage centers of The 34 Military Hospital Accident and Emergency Department and The Police Training School. At the triage center data entry clerks compiled the medical records of all suspected EVD cases. An EVD suspected case is defined as a person with acute onset of fever > 38 °C with any of the following additional symptoms: severe headache, muscle pain, vomiting, diarrhoea, abdominal pain, or unexplained haemorrhage; and had a direct contact with a suspected/confirmed EVD case or has unexplained multisystem illness that has been tested negative for malaria [21]. Later, all suspected EVD patients were transferred to the EHC for temporary admission while they were awaiting their EVD laboratory test results. Confirmed EVD patients were categorized into: Stage One (early phase) EVD patients that were febrile and presented with no vomiting, diarrhoea, or organ dysfunction at the time of admission; Stage Two (wet phase) EVD patients presented with vomiting or diarrhoea; and Stage Three which predominantly feature haemorrhage and organ dysfunction [22]. We considered EVD patients who were released alive after treatment as successful, those who died are said to have failed treatment. We did not account for both Ebola viral load or for the delay in seeking EVD treatment. The datasets generated and analyzed in this study were aggregate datasets that are being protected by the Sierra Leone Ethics and Scientific Review Committee in order to protect the identity of the patients whose medical data were analyzed.

### Treatment protocol

Both The 34 Military Hospital and The Police Training School ETCs medical personnel provided oral rehydration salts (ORS) and intravenous therapy (IVT) which included normal saline, lactated ringers, dextrose in water, and nutritional supplements to their EVD patients. The objective of these treatments was to correct for electrolyte imbalance. The key determinant of the ORS dose and IVT frequency was the degree of dehydration of the EVD patient. Additionally, the pain drugs acetaminophen or ibuprofen, the antibiotics ciprofloxacin or cefixime, and the anti-malaria tablets naphthoquine phosphate were provided to the EVD patients irrespective of their malaria status. The antacid drugs ranitidine or omeprazole were also provided to EVD patients who were experiencing lower abdominal pain. These drugs were administered either orally or intravenously to the EVD patients. These treatment protocols practiced at our study sites during the 2013–2016 EVD outbreak were the same throughout the period under investigation. These treatment regimens were performed in accordance with the World Health Organisation (WHO) protocol of urgent interim guide for EVD case management for viral haemorrhagic fever for 2013–2016 EVD outbreak [21]. The treatment protocols in the other Sierra Leone ETCs operating during the 2013–2016 EVD outbreak practiced mostly supportive care aimed at maintaining electrolyte balance by providing ORS to EVD patients.

### Statistical analysis

All data analysis in this study were done using R software package version 3.3.1 [23]. In this study, *p*-values < 0.05 were considered significant for all two-sided statistical tests. The outputs of our descriptive analysis were presented as frequencies, proportions, means and standard deviations (for continuous variables if they are normally distributed); medians and interquartile ranges (for continuous variables that are not normally distributed). Chi square tests were used to compare proportions of categorical variables. We used both univariable and multivariable logistic regression analysis to identify the clinical and non-clinical characteristics of EVD patients that were associated with EVD in-facility mortality. The Pearson’s correlation coefficient was used to determine the relationship between age and CFR. We later checked our crude mortality model for multicollinearity using Variance Inflation Factor (VIF) and any predictor with a VIF value above two was omitted from the adjusted mortality model. The smallest possible value of VIF is one. A VIF value that exceeds 5 or 10 indicates a problematic amount of collinearity [24].

We then constructed an Area Under the Curve (AUC) from the Receiver Operating Characteristic Curve (ROC curve) in order to determine the discriminating capacity of our adjusted mortality model to discriminate between EVD patient who will be released alive as compared to those who die during treatment given certain clinical and sociodemographic characteristics. To internally validate our predictive mortality model, we used the R package broom and bootstrap method with 1000 repetitions and re-sampling without replacement. We initially obtained the Area Under the Curve Original (AUC_Original_) for our multivariable logistic regression model as well as the Area Under the Curve for the bootstrap-corrected (AUC_corrected_) model. We then determined the performance of our predictive model by calculating the Area Under the Curve Optimism (AUC_optimism_) by subtracting the AUC_original_ from the AUC_corrected_.

## Results

### Ebola patient characteristics

**Fig. 1:**
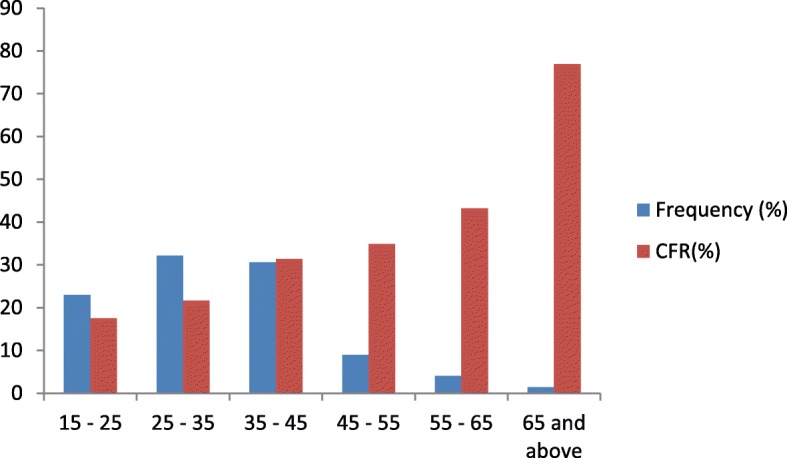
Distribution of EVD patients treated between the period of 2014–2015 at The 34 Military Hospital and The Police Training School ETCs during the 2013–2016 West African Ebola outbreak according to their age groups and respective CFRs

Out of the 938 EVD patients whose medical records were analysed, majority were males (59.0%, *n* = 553/938) and had secondary school education (79.3%, *n* = 744/938). The majority of the EVD patients belonged to age groups 25 years to 35 (32.1%, *n* = 301/938), and 35 years to 45 (30.6%, *n* = 287/938) (Fig. 1). The median age of our cohort group was 33 years (interquartile range = 25–40 years).

**Fig. 2:**
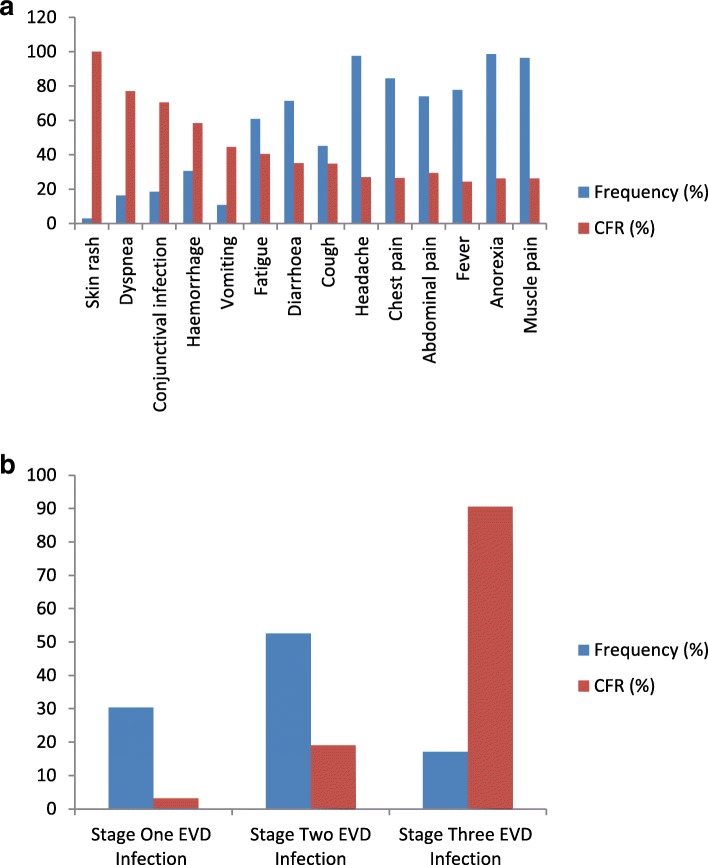
**a**. Distribution of the frequency of clinical symptoms of EVD patients treated between the period of 2014–2015 at The 34 Military Hospital and The Police Training School ETCs during the 2013–2016 West African Ebola outbreak and their CFRs. **b**. Distribution of the Stage of EVD Infection of EVD patients treated between the period of 2014–2015 at The 34 Military Hospital and The Police Training School ETCs during the 2013–2016 West African Ebola outbreak and their CFRs

Fever (77.7%, *n* = 729/938), headache (97.6%, *n* = 915/938), anorexia (98.7%, *n* = 926/938), muscle pain (96.5%, *n* = 905/938), chest pain (84.5%, *n* = 793/938), abdominal pain (73.9%, *n* = 693/938), diarrhoea (71.4%, *n* = 670/938), and fatigue (60.9%, *n* = 571/938) were the most reported symptoms at admission. The overall CFR recorded in this study was 26.4%. We recorded different CFRs and conducted bivariate analysis for the different clinical symptoms reported by EVD patients. Skin rash (CFR = 100.0%, *n* = 26/26, p = < 0.001), dyspnea (CFR = 77.1%, *n* = 118/153, p = < 0.001), conjunctival injection (CFR = 70.5%, *n* = 122/173, p = < 0.001), dysphagia (CFR = 68.5%, *n* = 196/286, p = < 0.001), and haemorrhage (CFR = 58.4%, *n* = 59/101, p = < 0.001) recorded high CFRs with significant association. Vomiting (CFR = 44.6%, *n* = 214/480, *p* = 0.01), fatigue (CFR = 40.6%, *n* = 232/571, p = < 0.001), diarrhoea (CFR = 35.1%, *n* = 235/670, p = < 0.001), cough (CFR = 34.8%, *n* = 147/423, p = < 0.001), abdominal pain (CFR = 29.4%, *n* = 204/693, p = < 0.001), and fever (CFR = 24.3%, *n* = 177/729 *p* = 0.006) recorded low CFRs with significant association. The associations between anorexia (CFR = 26.2%, *n* = 243/926, p = 0. 39), muscle pain (CFR = 26.2%, *n* = 237/905, *p* = 0.48) and chest pain (CFR = 26.5%, *n* = 210/793, *p* = 1.00) and CFR were not significant (Fig. 2a).

The clinical symptoms that are less frequent have high CFRs compared to those clinical symptoms that are common.

There were more (52.6%) Stage Two EVD patients compared to either Stage One or Stage Three EVD patients. Patients with Stage Three EVD infection (CFR = 90.6%, *n* = 145/160, p = < 0.001) recorded higher CFR compared to patients with Stage Two EVD infection (CFR = 19.1%, *n* = 94/493 p = < 0.001) or Stage One EVD infection (CFR = 3.2%, n = 9/285, p = < 0.001) (Fig. 2b).

**Fig. 3:**
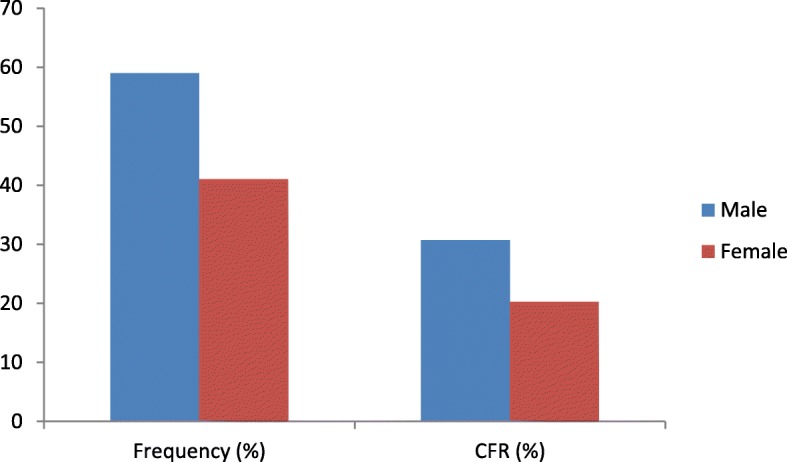
Distribution of EVD patients treated between the period of 2014–2015 at The 34 Military Hospital and The Police Training School ETCs during the 2013–2016 West African Ebola outbreak according to CFR and sex

The associations between CFR, sex (*p* = 0.0005), age groups (p = < 0.00001) and occupational levels (*p* = 0.0008) was statistically significant. The CFR for male EVD patients (30.7%, *n* = 170/553) was higher than female EVD patients (20.3%, *n* = 78/385) (Fig. 3).

Generally, the majority of the EVD patients were men and they also recorded higher CFR compared to women.

There was a positive correlation between age groups and CFRs; the CFR for the age groups 15 years to 25, 25 years to 35, 35 years to 45, 45 years to 55, 55 years to 65, and 65 years and above, were 17.5% (*n* = 38/217), 21.6% (*n* = 65/301), 31.4% (*n* = 90/287), 34.9% (*n* = 29/83), 43.2% (*n* = 16/37) and 76.9% (*n* = 10/13) respectively. Craftsmen (33.9%, *n* = 129/381) and nurses (28.6%, n = 10/35) recorded the highest CFRs amongst EVD patients with occupational record. The association between CFR and the education levels was not statistically significant (*p* = 0.13). For any increase in the education level there was a corresponding increase in the CFR; elementary (CFR = 22.8%, *n* = 18/79), secondary (CFR = 25.7%, *n* = 191/744) and tertiary (CFR = 33.9%, *n* = 39/115).

### Factors associated with in-facility EVD treatment mortality

We used multivariate logistic regression analysis to determine those EVD patients sociodemographic characteristics and clinical symptoms that are associated with in-facility EVD treatment mortality. Our stepwise multivariate logistic regression analysis following VIF check for multicollinearity shows that the Adjusted Odd Ratio (AOR) for EVD patients who were 65 years and above (AOR = 12.50, 95% CI = 2.32–80.74, *p* = 0.005) had increased odds of dying during treatment compared to EVD patients in the other age groups. Also, EVD patients who reported cough (AOR = 1.79, 95% CI = 1.12–2.86, *p* = 0.02), diarrhoea (AOR = 4.01, 95% CI = 1.85–9.40, *p* = 0.0008), vomiting (AOR = 3.21, 95% CI = 1.88–5.58, p = < 0.00001), fatigue (AOR = 2.64, 95% CI = 1.38–5.29, p = < 0.00001), dysphagia (AOR = 7.16, 95% CI = 4.40–11.80, p = < 0.00001), dyspnea (AOR = 3.63, 95% CI = 2.07–6.46, p = < 0.00001), and conjunctival injection (AOR = 3.45, 95% CI = 2.02–5.94, p = < 0.00001) during admission time had increased odds of dying during treatment compared to those who did not report these symptoms during admission time. However, the association for EVD patients who reported fever upon admission (AOR = 1.46, 95% CI = 0.87–7.98, *p* = 0.16) had increased odds of dying yet not statistically significant (Table 1).

**Table 1:** The Adjusted Multivariate Analysis of In-facility Case Fatality Rates of EVD patients treated between the period of 2014–2015 at The 34 Military Hospital and The Police Training School ETCs during the 2013–2016 West African Ebola outbreak

Patient symptoms	Adjusted OR	95% CI	*P* value
Sex – Male Reference = Female	1.50	0.95–2.26	0.09
Reference age group = 15 to < 25 years			
25 to < 35 years	1.09	0.58–2.38	0.80
35 to < 45 years	1.84	0.98–3.49	0.06
45 to < 55 years	1.64	0.70–3.90	0.26
55 to < 65 years	2.81	0.91–8.27	0.07
65 years and above	12.50	2.32–80.74	0.005
Fever	1.46	0.87–7.98	0.16
Cough	1.79	1.12–2.86	0.02
Vomiting	3.21	1.88–5.58	< 0.00001
Diarrhoea	4.01	1.85–9.40	0.0008
Fatigue	2.64	1.39–5.29	< 0.00001
Dysphagia	7.16	4.40–11.80	< 0.00001
Conjunctival injection	3.45	2.02–5.94	< 0.00001
Dyspnea	3.63	2.07–6.46	< 0.00001

^a^Adjusted OR is Adjusted odds ratio

**Fig. 4:**
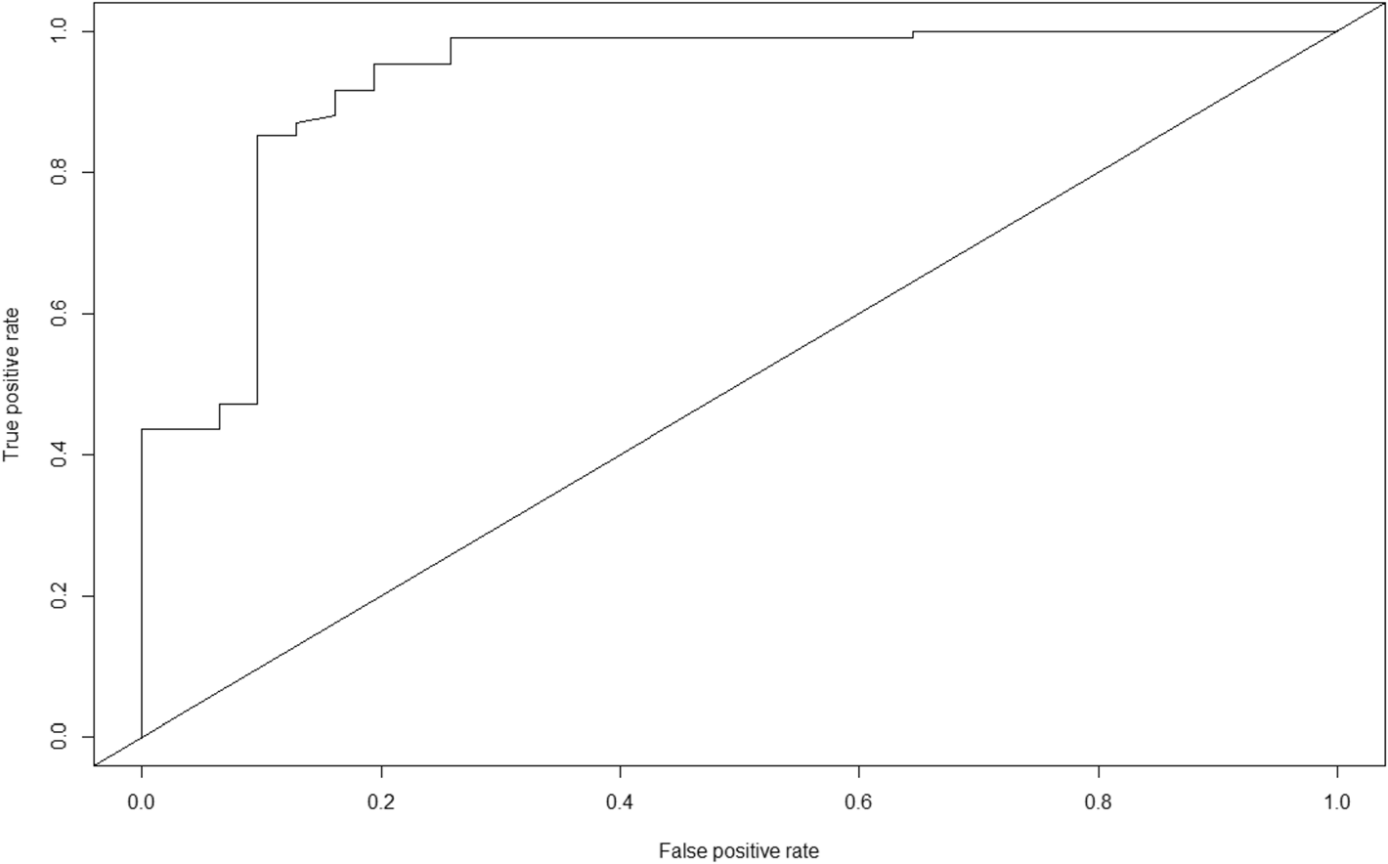
ROC Curve on EVD Treatment Survival Determinants and Treatment Outcome

To internally validate our multivariate logistic model, we calculated the mean optimism of our model by subtracting the AUC value of our optimism-corrected ROC curve from the AUC value of our multivariate (original) ROC curve and multiplied it by 0.5. The Akaike Information Criterion (AIC) value of the ROC curve for our multivariate (original) model produced an AUC_original_ of 0.935 (Fig. 4) while our optimism-corrected AUC (AUC_correctedoptimism_) for our final (adjusted) model which included the age group 65 years and above, and EVD patients who reported fever, cough, vomiting, diarrhoea, fatigue, dysphagia, conjunctival injection, and dyspnea at the time of admission model was 0.932. Our mean optimism [(AUC_original_ – AUC_correctedoptimism_) × 0.5] is 0.002.

Using the clinical and demographic characteristics of our EVD patients and the ROC curve, our final model has the capacity to discriminate their treatment outcomes.

Using an arbitrary threshold of 0.945 to prioritize an optimal positive predictive value for fatal outcome, our model successfully identified 79.4% (Sensitivity = 197/248) of those EVD patients who actually died during treatment (Table 2). Our sensitivity, specificity, positive predictive value and negative predictive value of our model are 79.4% (197/248), 100% (690/690), 100% (197/197) and 93.1% (690/741) respectively.

**Table 2:** Matrix of actual and predicted treatment of outcome for EVD patients

EVD Patients predicted status	EVD treatment survivors	EVD treatment fatalities	Total
Predicted EVD survivors	690	51	741
Predicted EVD fatalities	0	197	197
Total	690	248	938

## Discussion

We analyzed the aggregated medical information of adult EVD cases who registered for treatment at our study sites in order to determine the CFRs for the various subsets of these patients. We believe that the clinical characteristics and treatment outcomes of adult EVD cases are likely to be different and non-linear along age groups if adults and children are combined as well as for the various subsets of adult EVD cases. Different values of CFRs for different locations and settings [10–13, 25] which can be attributed to the subpopulations investigated and pre-selection biases, [10, 25] were reported during the 2013–2016 EVD outbreak. We reported a low CFR (26.4%) for EVD cases treated at The 34 Military Hospital and The Police Training School ETC in Sierra Leone compared to the CFR computed by Wong et al. and the WHO for the same outbreak in Sierra Leone were 74.2% (95% CI: 72.6–75.5%) [24] and 28% (3956/14124) [26] respectively. We may attribute the reasons for our low CFR to the type of professional health care that was provided in a well-staffed and equipped military facility in a capital city setting, and to the treatment regimen used in our ETCs compared to those implemented in other ETCs in the country.

Specifically, unlike other ETCs that only administered ORS, nutritional supplements, antiemetic, fever and pain relieving drugs to their EVD patients during the EVD outbreak in Sierra Leone [27], the medical personnel at The 34 Military Hospital and The Police Training School ETCs administered ORS, IVT, and other medications at different rates to their EVD patients throughout the outbreak period. Intravenous parenteral drug administration provides an easy and rapid drug administration leading to immediate drug action. It also enable drugs to be administered either continuously or intermittently thereby resulting into rapid changes in the cardiocirculatory system [28], increase in blood and plasma volumes [28–30] and makes the monitoring of the delivered fluids, electrolytes and nutrients easier. Generally, for EVD patients at the various stages of infection, the loss of bodily fluid, electrolytes and nutrients are the major clinical effects of the disease. Replacing lost bodily fluids effectively and rapidly via IV drug administration is a life saver for EVD patients [30].

One unique feature of the treatment regimen offered to EVD patients in our study is the IV administration of the antimalarial drug; artesunate 120-mg once daily for 3 days irrespective of their malaria status. Sierra Leone is a malaria endemic country; malaria peaks during the period in which this study was conducted. The high malaria transmission during the study period raised the suspicion of occult malaria infection and hence warranted the administration of such drug. Many studies have reported different treatment outcomes for EVD patients co-infected with malaria [31–37] hence treating patients for EVD and malaria simultaneously will invariably improve their prognosis.

Other reasons for these CFR variations may include incomplete EVD case ascertainment, thoroughness of reporting EVD clinical outcome, and the epidemiological case definitions used [25]. Our failure to account for the delay in seeking EVD treatment outcome following onset of signs and symptoms however implies our cases may not have been true representative of the national characteristics of reported EVD cases. Generally, studies that are less representative produce CFR estimates that vary with the national estimate and are often considerably lower [8, 19].

Our high CFR and AOR associated with nurses, craftsmen and unemployed patients which may be attributed to EVD over exposure. Healthcare workers have been specifically linked with high EVD incidence and CFR [17, 38–40] due to occupational exposure and the nonspecific clinical symptomology of EVD [41, 42]. This nonspecific clinical symptomology of EVD makes it difficult for healthcare workers to differentiate it from other tropical febrile infections during the early phase [42] of an outbreak resulting to delay in seeking EVD treatment and hence leading to its high CFR and AOR. The high CFR and AOR for both craftsmen (auto mechanics, electricians, farmers, truck pushers, mine workers, hunter, builder and carpenter) and unemployed EVD patients can be attributed to their unstable risky living conditions and high mobility which can potentially lead to over-exposure. Public health education is important in understanding EVD’s signs and symptoms [43, 44], evolution and mode of transmission which enables people to seek early treatment. Levy B et al. have previously linked the severity (CFR) of an EVD outbreak to the level of prior knowledge and health education of the general population [45]. Additionally, we may also want to associate our low CFR to the large number of EVD patients with secondary and tertiary levels of education. Although we observed a positive association between education levels, age groups and CFRs which may be associated with the increasing number of old EVD cases as one progresses from one education level or age group to the other, we believe that the large number of EVD patients with both levels of education in our study could have been the major factor for this low CFR. Educational attainment influence treatment mortality through other dimensions including access to treatment and seeking treatment early. Our low in-facility CFR may in fact refer to a pre-selected subgroup of patients, and a-priori excludes an important group of EVD-cases that either does not make it to an ETC, or that are not admitted due to limited sensitivity in the employed case definitions. Our study can thus be used to assess and evaluate the efficacy of our EVD treatment methods as well as to compare it with others in different parts in Sierra Leone. However, the fact that our CFR is based on in-facility data presents a challenge, since external validity towards settings outside an ETC is limited.

Our study report clinical symptoms similar to those reported in other EVD studies [8, 9, 19] but which are also common to other tropical infections including malaria, yellow fever, dengue, cholera or Lassa fever. We recorded a higher prevalence and CFR for EVD cases with gastrointestinal symptoms (diarrhoea and vomiting). Gastrointestinal symptoms often lead to electrolyte abnormalities including hypokalemia, hypoglycemia, and hypocalcaemia that were common clinical presentations during the 2013–2016 West Africa EVD outbreak [9, 14, 19, 46]. We observed that EVD patients with diarrhoea had increased odds of dying during EVD treatment than those without it. Our high CFR for diarrhoea and vomiting may be associated with their roles on several metabolic abnormalities including metabolic acidosis and alkalosis. Diarrhoea and vomiting can cause hypovolemic shock and metabolic hyperchloraemic acidosis through dehydration [47]. Schieffelin JS et al. had previously reported the presence of acidosis and elevated blood urea nitrogen and creatinne as predictors for EVD diagnosis and fatality [12]. Our statistically significantly higher CFR values and odds ratios for both diarrhoea and vomiting indicates that these clinical features should be recognized during EVD screening, patient management and transmission control mechanism during early EVD outbreaks. The majority of the EVD cases in our study had fever, headache, anorexia, muscle pain, chest pain, abdominal pain, diarrhoea, and fatigue which are consistent with studies by Bah et al [43], Mupere et al. [48], and Theocharopoulos et al. [49] Bah et al. reported a 43.0% CFR in their study in which majority of the study participants had fever (84.0%), fatigue (65.0%), and diarrhoea (62.0%) [43]. Mupere et al. reported more than 50% of EVD cases presented with either fever, headache, weakness, anorexia, diarrhea, or vomiting at the time of admission [48]. The most common symptoms for 249 EVD cases with a CFR of 45.0% reported by Theocharopoulos G and colleagues were malaise (90.0%), fever (83.0%), diarrhoea (63.0%), headache (73.0%) and vomiting (60.0%) [49].

One challenge in the early detection of EVD cases in resource poor settings is the similarity of its clinical symptoms to that of other tropical infections. This similarity makes the use of a single EVD symptom checklist inadequate in outbreak foci; and hence calls for EVD case definition criteria with higher discriminatory capacity during early outbreak period. Such an EVD outbreak case definition tool is needed especially in settings with both logistical challenges and high risk of nosocomial EVD transmission and during the early EVD outbreak phase; to differentiate non-EVD patients from confirmed, suspected or probable EVD cases. Any tool that can make use of the clinical symptoms and sociodemographic characteristics contained in our final model to identify those confirmed EVD patients with high risk of dying during the early phase of an EVD outbreak will reduce the CFR as well as ensures the diversion of much needed logistics to other areas of EVD case management, control and prevention. EVD outbreaks in Low- and Middle-Income Countries usually occur in remote communities with poor road network and limited or no laboratory facilities. Blood samples from confirmed, suspected or probable EVD cases usually had to be transported long distances to bigger towns for laboratory tests; all of which increase the delay in seeking EVD treatment and CFR. Mupere E et al. had earlier proposed an EVD case definition to include the categorization of risk into EVD suspected, probable or contact cases [48]. Such EVD risk categorization if applied to EVD case definition lacks the descriptive specificity for a clinically useful case definition and hence cannot be incorporated for widespread use during EVD outbreak. Our high AUC (0.935) which quantitatively discriminate between EVD patients who were treated and released alive from those who died during treatment has both clinical and prognostic relevance which allows the best possible identification of patients in need of the usually scarce resource of intensified attendance. Given the limited availability of EVD treatment logistics during EVD outbreaks in resource-constrained settings, the allocation of resources (medical attention, materials, bed space) to individuals identified as high-risk patients at the time of admission through the use of algorithms stipulated by our model could have predicted with 100 and 93.1% accuracy these patients as dying or surviving in the past outbreak respectively. Our model will be equally useful where the safety of patients with respect to avoiding nosocomial EVD infections within healthcare facilities is a dominant concern.

Because this is a retrospective study in which we only analysed data that have been previously collected, we were not able to determine the effect viral load had on the treatment outcome of an EVD patient; our medical record did not capture such variable. This limitation only permitted us to determine the onset of EVD by the clinical signs and symptoms of the EVD patient and not by the presence of Ebola Virus viral load. Another limitation is the unavailability of data on presented but unconfirmed patients. The comparison to this group would have allowed for a differentiated analysis of clinical presentation between confirmed and unconfirmed patients. Additionally, we did not follow up EVD patients who were released alive following treatment in order to determine the factors that may be associated with late post-release mortality. An important finding from these follow up visits would have been to conduct a comparison between confirmed EVD and non-EVD patients alongside their calculable attributable risks. The lack of inclusion of non-EVD patients in our data base who may have suffered from substantial morbidity and mortality collaterally to EVD should thus receive considerably attention in future settings. Another challenge within the clinical context of our study was the difficulty in providing adequate clinical care for all patients within our treatment facilities as well as how to keep track and ensure that they receive the therapies they were supposed to receive. We also believe that in spite of these limitations our low CFR may have also been due to the fact that our patients were admitted to a military hospital which was supported with better resources that may not have been present in other ETCs operating in the country during the outbreak.

Additionally, our study may not be generalized to the entire Sierra Leone population at the time because the Western Area where this study was conducted benefited from a lot of community engagement and educational campaigns which may have affected the access and transfer of EVD patients to our study sites. These community engagement and educational campaigns may have also led to the less prevalence of EVD-related stigma compared to other areas in the country.

## Conclusion

We constructed a simple CFR risk classification system that can identify EVD in-facility treatment mortality predictors based on the sociodemographic characteristics and clinical symptoms of the EVD patient. We also reported low EVD cases among patients with secondary and tertiary education. These subpopulations of our patients who are generally informed about the signs and symptoms of EVD, alongside our treatment regimen may have been responsible for our comparatively lower case fatality rate.

## Data Availability

The data that support the findings of this study are available from The 34 Military Hospital in Sierra Leone but restrictions apply to the availability of these data, which were used under license for the current study, and so are not publicly available. Data are however available from the authors upon reasonable request and with permission of Sierra Leone Ethics and Scientific Review Committee, and The 34 Military Hospital in Sierra Leone which was managing the ETCs at The 34 Military Hospital and The Police Training School.

## Abbreviations

AIC: Akaike Information Criterion
AOR: Adjusted Odd Ratio
AUC: Area Under the Curve
CFR: Case Fatality Rates
EHC: Ebola Holding Center
ETC: Ebola Treatment Center
EVD: Ebola Virus Disease
IVT: Intravenous Therapy
LMU: Ludwig-Maximillian’s Universidad
NERC: National Ebola Response Center
ORS: Oral Rehydration Salts
ROC: Receiver Operating Characteristic Curve
RT-PCR: Reverse Transcriptase Polymerase Chain Reaction
SLAF: Sierra Leone Armed Forces
WHO: World Health Organisation
VIF: Variance Inflation Factor
